# Exploring Chemoinformatics Aspects of Few-Shot Meta-Learning by Example of an Infinite Dilution Activity Coefficient in Ionic Liquid Prediction

**DOI:** 10.1021/acs.jcim.6c00067

**Published:** 2026-04-13

**Authors:** Karol Baran, Adam Kloskowski

**Affiliations:** Department of Physical Chemistry, Faculty of Chemistry, 431446 Gdansk University of Technology, Narutowicza Str. 11/12, 80-233 Gdansk, Poland

## Abstract

Collection of substantial training sets for structure–property modeling often poses a significant challenge, especially for niche sorption systems. This study provides computational experiments with meta-learning that presents a compelling solution to the data scarcity challenge in molecular property prediction. Our analysis utilized infinite dilution activity coefficients for several ionic liquid–solute systems, with coefficient prediction for systems with a particular solute constituting a task. The systems were modeled predominantly using model-agnostic meta-learning (MAML), supported by a study on Reptile and its modified variants. The obtained results provide promising insights into training MAML models by expanding the adaptation set size. Metrics such as *R* ^2^, RMSE, and MAE indicate comparable performance to graph neural networks, even when trained on only 64 or 128 data points. The versatility of the fine-tuned models suggests that, in certain cases, performance on a single task may be achieved at the expense of reduced model versatility (catastrophic forgetting). The similarity between the test and training tasks (as approximated by Tanimoto similarity of the solutes’ molecules) was identified as a factor affecting performance on the test task. Consequently, Task Similarity-Aware Reptile (TSA-Reptile) was proposed to target those dissimilar tasks. This novel method scales the loss function by similarity to the closest training task. It was shown to outperform MAML on out-of-distribution tasks. Beyond providing the comparative analysis of meta-learning and traditional deep learning, potential strengths of both MAML and TSA-Reptile are discussed.

## Graphical abstract

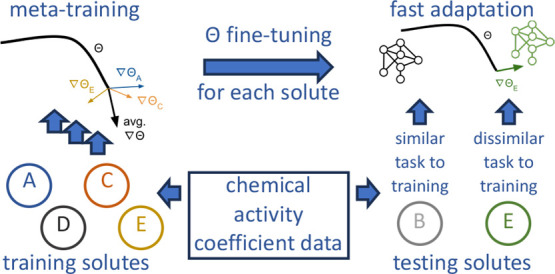

## Introduction

The evolution of chemistry is increasingly influenced by theoretical insights and computational methodologies, which illuminate the molecular behavior. These tools surpass conventional experimentation, enabling researchers to predict, analyze, and design chemical systems with a precision. Computational power and theoretical models allow researchers to explore vast chemical possibilities, uncovering patterns and principles that enhance our understanding of the natural world. This integration enhances scientific inquiry and broadens the discovery across the field of chemistry.

Computational approaches in chemistry play a pivotal role in guiding experimental endeavors by identifying the most promising compounds for exploration. Computer-aided methods are gaining prominence due to their ability to mitigate the challenges associated with scale, time, and cost inherent in traditional experimental techniques.^1^ By facilitating the simulation and analysis of intricate systems, these methodologies offer insights into reaction mechanisms, solvent effects, and material properties that are often challenging or expensive to determine experimentally. Their applications span physical chemistry, environmental studies, and solvent selection, contributing to more sustainable practices and an enhanced theoretical comprehension.^2^ In the context of studying interactions between solvents and solutes, activity coefficients, particularly at infinite dilution (IDAC), hold a significant importance. In chemical engineering, they facilitate the modeling of the phase behavior and transport processes. Activity coefficients aid in predicting chemical potentials and system behavior in both binary and multicomponent mixtures, rendering them indispensable for the process design and optimization. Their prediction through computational techniques is an extensively explored subfield.^3^ Consequently, computational techniques increasingly complement and accelerate discovery in both fundamental and applied chemical sciences.^2^

Chemoinformatics is an interdisciplinary field that specializes in employing computer science techniques to address challenges within the field of chemistry.^4^ One of the most fundamental concepts in this field is chemical space, which frequently refers to ensembles of potential molecules that could theoretically be of interest for various applications in drug discovery or chemical engineering.^5^ Structure–activity relationships may exhibit nonlinearity or discontinuity, as structurally similar compounds or analogues can show significantly different potencies against the same biological target.^6^ Navigating this vast space presents both challenges and opportunities. Identifying promising candidates necessitates efficient methods for molecular representation, comparison, and property prediction.^4^

Molecular property prediction (MPP) is a fundamental task in chemoinformatics. It enables the identification of more suitable molecules for specific applications, both expediting and cost-effectively, while ensuring reasonable accuracy.^7^ The objective is to correlate chemical structure and corresponding property value. To achieve this objective, sophisticated machine learning (ML) algorithms are employed. To study molecular and multimolecular systems, like those that contain compounds like ionic liquids (ILs), several ML algorithms have been applied in quantitative structure–property modeling (QSPR).^7–9^

In contrast, in recent years, researchers have increasingly focused on neural networks (NNs), particularly in the field of graph neural networks (GNNs).^9^ These algorithms fall under the broader category of deep learning (DL) techniques. Unlike traditional ML algorithms that process tabular data in the form of molecular descriptors, DL algorithms employ raw data representations, such as SMILES or molecular graphs.^10^ Advancements in DL in other computer science applications^11^ hold promising prospects for chemoinformatics as well.^12^ A study by Sypetkowski et al. demonstrated significant performance improvements in DL models as the model size, data volume, and available computational resources increased. Models with a larger parameter count, particularly the 3-billion-parameter versions of graph-based and transformer neural networks, exhibited consistent gains on the original test set and in subsequent fine-tuning tasks.^13^

The primary limitation of DL methods is their substantial data requirements for training. This limitation is partially mitigated by the use of meta-learning techniques that enable pretraining of NNs on data sets covering analogous problems (tasks), followed by adaptation on data sets corresponding to the target task.^14^ These techniques are sometimes termed few-shot learning, as they enable the construction of useful models by adapting to a limited number of data samples.^15^ One of the recently investigated algorithms in this field is model-agnostic meta-learning (MAML), whose learning objective is to train a model to quickly adapt to novel tasks.^16^ MAML-based models are believed to yield satisfactory performance despite utilizing a relatively small adaptation set in conjunction with deep neural network architectures.^17^ However, the appropriate application of algorithms such as MAML in the field of MPPs requires evaluation in a chemical context.

## Related Works

The MAML algorithm has previously been shown to facilitate low-data learning in molecular property prediction. Dou et al. identified MAML as one of the algorithms that enable low-data machine learning in chemical applications.^18^ In a separate study, Ju et al. compared MAML to other meta-learning techniques for MPP.^19^ In a study conducted by Pappu et al., the researchers investigated the application of few-shot learning techniques utilizing both GNNs and molecular fingerprints. While GNNs generally performed inferiorly to fingerprint-based methods, incorporating meta-learning techniques, such as MAML, further improved their performance. Notably, MAML surpassed both pretraining and fingerprint-based approaches in low-data scenarios.^14^

Nguyen et al. investigated whether MAML could enhance the performance of DL methods in low-data regimes. They discovered that metainitializations enabled superior performance compared to other DL methods. The study compared the MAML model with a *k*-nearest neighbors (*k*-NN) trained on GNNs embeddings and a fine-tuned GNN. Twenty-three test tasks were selected from the total of 906 for model testing. The majority of the tasks were drawn from the CHEMBL data set, although some included toxicity prediction or physicochemical property prediction. The analysis indicated that metainitialization improved model performance and concluded that their research could facilitate the application of DL to chemical data sets with limited training instances.^20^ Meng et al. conducted an evaluation of task augmentation and the commonalities among various tasks. They observed that few-shot MPP is a less-studied problem despite its difficulty and its significant importance for increasing success rates in virtual screening. After identifying MAML as the most promising algorithm, they investigated how metainitialization could enhance MPP modeling. They identified two primary risks associated with meta-training: the potential for the classifier to memorize all query samples or the learner to overfit to training tasks, resulting in inadequate adaptation for test tasks.^21^

Guo et al. proposed a protocol wherein, for each task, a small training set is utilized to update the GNN, which is subsequently evaluated on a distinct query set. This iterative process is repeated across tasks, thereby improving model performance. Furthermore, a self-supervised module facilitates the GNN’s learning process by predicting atom types and bond presence. Additionally, a task-aware attention mechanism assigns task-specific weights based on their molecular representations, thereby optimizing model updates.^22^ Kötter et al. conducted a study to assess the impact of providing comparable data in meta-training on the performance of fine-tuned models based on biological activity data.^23^ In a comparative study by Lv et al., the authors evaluated the performance of MAML against DL methods on a data set of quantum mechanical parameters of molecules (regression). The findings demonstrated MAML’s superior performance in this specific task.^24^ In other research, Lv et al. evaluated MAML among several few-shot algorithms for predicting drug–drug interactions. MAML demonstrated upper-level performance among the evaluated algorithms.^25^ Qian et al. conducted a study on MAML, an attention-based GNN pretrained on CHEMBL data sets, to enhance classification performance. Their research focused on using an attention mechanism to generate task-specific embeddings for compounds within the same task. In this system, MAML demonstrated superior performance compared to other few-shot techniques on the MoleculeNet benchmark, as reported in ref 26.

In the literature on the subject, there are limited works on applying MAML to sorption problems, particularly involving ILs. Furthermore, the comparison between DL and emerging MAML modeling is not a commonly discussed topic, primarily because the scope of the task definition often constrained the problems investigated in previous studies. However, applying MAML to activity coefficient prediction offers an opportunity to explore both DL and meta-learning perspectives. This approach offers valuable insights into MAML’s added value. Given that, in the context of solvent–solute interactions, each task is defined by the chemical type of the solute, it is possible to approximate task similarity based on solute molecular similarity, for example, using Tanimoto similarity. This intuition can be used to quantitatively evaluate MAML performance based on task similarity. Theoretically, applying MAML to this area should be an intriguing research topic. One could meta-train a model on a large data set of chemical compounds and adapt (fine-tune) it for specific needs, eliminating the need to recalibrate the entire model for adapting it to novel solutes. Additionally, DL models may perform poorly on niche tasks. This limitation should be potentially mitigated by a two-stage protocol enforced by the MAML design. In light of the rationale mentioned above, the research questions posed in the current research are as follows.

1. Is the convergence of the MAML meta-trained model slower than that of traditional learning protocols, as exemplified by the data set on activity coefficient prediction for IL–solute systems?
2. Does the MAML model deliver comparable performance to the model generated using DL with a conventional learning protocol on test tasks (particularly those that are novel to the model) after adaptation?
3. How much data is required for adaptation of the MAML model in a few-shot manner based on the case study of IDAC in the ILs benchmark?
4. Is model adaptation associated with a decrease in the model’s performance on other tasks? Is catastrophic forgetting a risk during the adaptation process?
5. Is there a strategy to select an adaptation set that maximizes the model’s performance?
6. Does task similarity (measured as the molecular similarity of solutes) impact the quality of the model after the adaptation?

## Methods

### Data Set and Problem Definition

The data set on infinite dilution activity coefficients available in the literature of the topic^27,28^ was selected for this study. The selection of this data set was guided by several key motivations relevant to the study’s objectives. The data set focuses on ionic liquids, which have significant scientific relevance in structure–property modeling due to their tunability, enabling the design of solvents with tailored properties.^29^ Furthermore, the data is freely accessible from the ILThermo database, facilitating reproducibility and transparency.^30^ Notably, this data set has been previously employed successfully in deep learning applications, underscoring its utility and robustness. Its substantial size provides a meaningful foundation for exploring GNN and MAML modeling approaches. Importantly, each solute in the data set can be considered a distinct task, aiming to predict the infinite-dilution activity coefficient for ionic liquids that have not yet been studied in systems containing that solute. This structure allows the problem to be addressed using conventional GNN methodologies, thereby enabling direct comparisons with meta-learning techniques. Additionally, the data set comprises approximately 120 tasks spanning a broad spectrum of chemically related, highly diverse systems, as elaborated in subsequent sections. The data set was presplit into training and testing subsets based on IL–solute combinations.^27^ In this study, this predefined split is still incorporated. However, greater attention is paid to individual tasks (solutes) in accordance with meta-learning principles. Consequently, both predefined training and test splits are assigned an appropriate task ID, enabling the selection of tasks for meta-testing as described in the subsequent sections. Instant JChem was used for data storage and management, Instant JChem v. 24.3.1, Chemaxon (https://www.chemaxon.com).

### Data Preprocessing

In this study, the data set was divided into training and test sets, following the protocol established in previous research.^27^ This approach was adopted to maintain consistency and ensure comparable results across studies. The data split was designed to prevent the same IL–solute combinations from appearing in both the training and test sets. This methodology ensures that during the testing phase, the model is evaluated on ILs that were not previously encountered during the adaptation of the MAML model. Such a strategy is crucial for assessing the model’s generalization capability to novel ILs.

The target IDAC on a logarithmic scale was used as the target variable. IDAC values are frequently studied after logarithmic transformation,^31^ but the precise value can be readily determined from the model prediction using the inverse power transformation.

Given the highly skewed data distribution and its span across multiple orders of magnitude, an additional transformation was required. The data were scaled by using a quantile transformation. This preprocessing step helps normalize the data distribution, making it more suitable for machine learning algorithms, which often perform better when the input data are uniformly distributed. To ensure stable neural network training, normalization to the range of 0–1 was implemented. Target transformations and scalers were fit strictly on the training data and applied to the full set. The transformations mentioned above were executed utilizing the scikit-learn library.^32^

Furthermore, the simplified molecular input line entry system (SMILES) representations of both cations and anions were normalized using the standard procedures available in the RDKit library.^33^ This normalization process ensures a consistent representation of molecular structures, which are critical for accurate feature extraction.

Molecular similarity calculations were performed using the Tanimoto similarity metric, which operates on Morgan fingerprints. These fingerprints were generated with a radius of 2 and a bit vector size of 2048 bits. This specific configuration was chosen to capture detailed structural information while maintaining computational efficiency. For each solute, the highest similarity value was identified to determine the nearest neighbor based on structural similarity. This nearest neighbor analysis provides insights into the structural relationships within the data set, which can influence the model’s predictive performance. All the data or model training-related graphics were rendered using the Matplotlib library.^34^

### Modeling Based on GNNs

Learning in GNNs involves updating the information stored in the graph structure by applying weights and normalization factors at each iteration. In essence, this process entails passing information from one node to another within the graph, thereby establishing a neural message-passing schema that is a more generalized version of the matrix convolution. Given that this optimization process aims to minimize model error, backpropagation is used to update the model weights. During backpropagation, the learning rate determines how conservatively weights are updated, reflecting the degree of weight refinement.

Drawing on the findings of our previous study, we decided to employ IL geometry optimization independently for each ion, subsequently merging the optimized geometries into a single graph without fictitious ionic bonds. Furthermore, we augmented this graph with information on formal charges. A separate graph was constructed for the solute and underwent its own convolution layers. Subsequently, the two embeddings, one for IL and the other for the solute, were concatenated with temperature just before the final linear layers. Notably, the same GNN architecture employed in this study remains unchanged. The details on model’s architecture followed our previous work.^9^ The hyperparameters selection with random search and additional details are provided in Appendix A (Supporting Information). The models were developed utilizing the PyTorch^35^ and PyTorch Geometric^36^ Python libraries. The used Atom Message Passing^37^ style function was reimplemented into PyTorch Geometric.

To assess the efficacy of the model, several performance metrics were employed, including the coefficient of determination (*R* ^2^), root-mean-square error (RMSE), and mean absolute error (MAE), which are defined in eqs 1–3

$$R^{2}=1-\frac{\sum_{i=1}^{n}(y_{i}-{\hat{y}}_{i}{)}^{2}}{\sum_{i=1}^{n}(y_{i}-\overset{-}{y}{)}^{2}}$$ 1

$$\mathrm{RMSE}=\sqrt{\frac{1}{n}\sum_{i=1}^{n}(y_{i}-{\hat{y}}_{i}{)}^{2}}$$ 2

$$\mathrm{MAE}=\frac{1}{n}\sum_{i=1}^{n}|y_{i}-{\hat{y}}_{i}|$$ 3

where *n* is the number of observations, *y*_*i*_ represents the actual values, *ŷ*_*i*_ represents the predicted values, and *y̅* is the mean of the actual values.

The evaluation metrics were assessed across all tasks, encompassing training, validation (10% of the training data), and test sets.

### Meta-Learning Modeling

Meta-learning is a machine learning technique whose objective is learning how to learn. The MAML algorithm is not trained on a specific task (e.g., predicting whether a solute will be soluble) but rather on a collection of diverse tasks (e.g., predicting solubility from multiple data sets for distinct solutes).

The model’s parameters are learned from a diverse range of tasks, enabling effective generalization to novel tasks with minimal training data. In contrast, while traditional machine learning emphasizes training a model to excel at a particular task, the objective of MAML is to acquire a model that may not necessarily perform optimally out of the box but rather can be readily adapted for various tasks.

MAML is trained in two-step protocol. During meta-training, the model is fed with a batch of tasks. For each task, a support set (consisting of 128 samples) is utilized to adapt the model in the inner loop. Each adapted model is evaluated on query sets in the outer loop. The episode of training concludes with update of the weights of the meta-model. After multiple episodes, the model is evaluated on meta-test tasks. The evaluation metrics are presented as averages across five sampling iterations of the adaptation set (with distinct random seeds). Besides the average metric value, the standard deviation was used as an uncertainty approximation. This assessment facilitates the evaluation of the model’s learning and adaptation capabilities for the specific task. Consequently, across all models, the model with the lowest loss on the validation set was selected. The hyperparameter selection with random search is explained in Appendix A (Supporting Information). The MAML model was coded utilizing the learn2learn library.^38^

To assess the model’s generalization capabilities, it is evaluated on meta-test tasks that it has not encountered before. In a highly simplified scenario, one could envision using IDAC data in systems containing water, ethanol, or pentanol for meta-training. Subsequently, data on IDAC in systems containing butanol could be employed for meta-testing. However, real-world applications necessitate significantly larger data sets. Of the approximately 120 tasks in the IDAC data set, 12 were selected for meta-testing. These tasks encompassed both similar tasks (6 tasks randomly selected from those with the highest Tanimoto similarity to the closest neighbor in the meta-training set) and dissimilar tasks (6 tasks randomly selected from those with the lowest Tanimoto similarity to the closest neighbor in the meta-training set). To ensure proper experimentation, only tasks with at least 256 training instances were used. Each of these tasks had its own support and query sets, allowing us to see how well the model performs in new situations with minimal data. The MAML model, as outlined in the preceding section, diverged from the GNN model primarily in its training protocol, while maintaining the same GNN architecture. The final evaluation of model performance is conducted per task using the subset of the data set preassigned the testing label in the preceding study.^27^ The difference between meta-training, meta-testing, adaptation, and testing is worth further clarification, however. The MAML model is meta-trained with portion of tasks that are called meta-training tasks. The holdout tasks, called meta-test tasks, are utilized for evaluation on how well the MAML model adapts to novel tasks. Consequently, they undergo adaptation with data labeled for training^27^ serving as the adaptation set. The query set consists of samples labeled for testing.^27^

Figure 1 presents the data partitioning protocol employed in this study. Chemical data is fundamentally heterogeneous, spanning diverse solutes, solvents, and experimental conditions. The complete data set, denoted as *D*, comprises tuples of the form (IL, *S*, *T*, and γ), where IL represents the chemical structure of the ionic liquid, *S* denotes the chemical structure of the solute, *T* indicates temperature, and γ is the infinite dilution activity coefficient. In alignment with the data source,^27^ the data set was split into *D*_train_ and *D*_test_ using a random split, ensuring no overlap in IL–*S* combinations. In the MAML-based modeling process, each solute *S* was treated as an individual task. The majority of the tasks were used for meta-training the model, while a subset of 12 tasks was designated for meta-testing, with 128 samples serving as the adaptation set (*D*_m_ad__ ∈ *D*_train_), and a portion of the task data allocated as the query set (*D*_m_q__ ∈ *D*_test_). This data-splitting strategy ensures that the MAML model, once adapted for a specific task, is evaluated on ILs that were not involved in the model’s adaptation phase. This distinction is crucial, as predicting the γ of a previously seen IL at a new temperature is significantly easier than predicting γ for entirely novel ILs.

**Figure 1:**
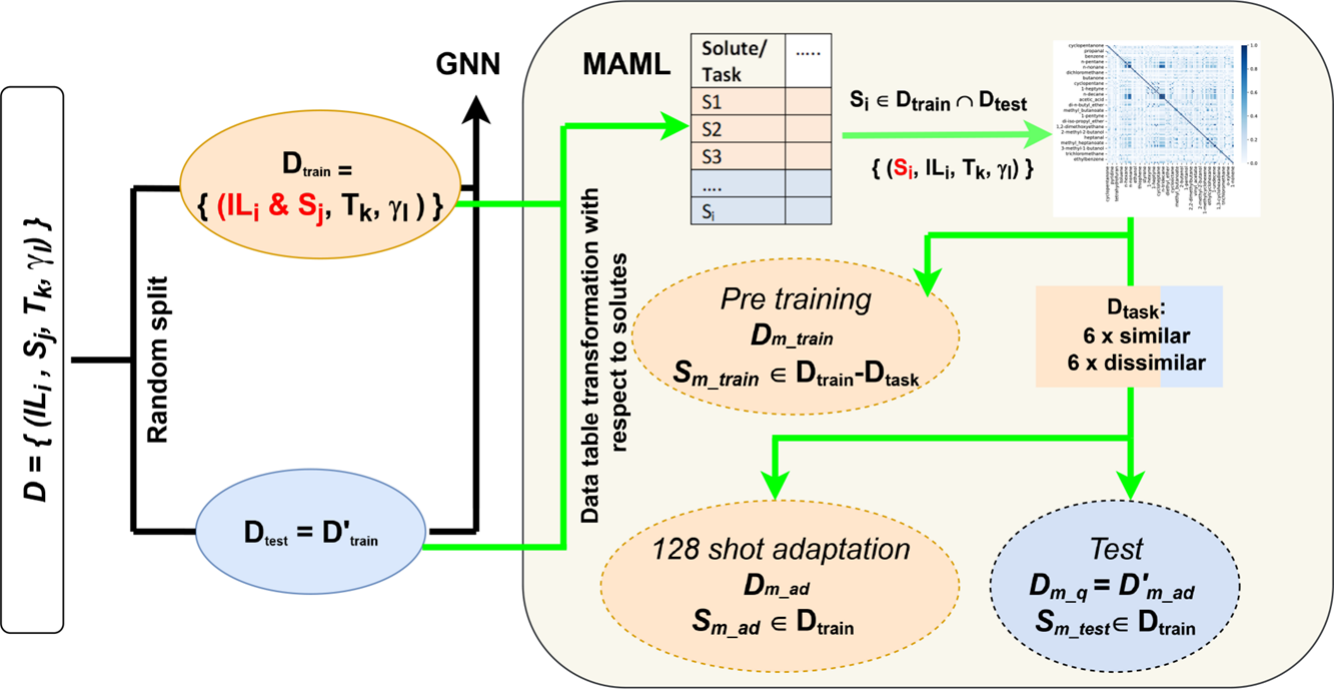
Data split protocol used in this study. The elucidation of the symbols furnished within the main text.

The comparison between GNN and MAML raises the need for a proper formulation of the problem. In the GNN training protocol, the full training set is utilized during training. Consequently, the model learns patterns that are visible from all tasks. In this particular case, this is not an obstacle, since the target variable is meaningful regardless of the task definition. In other words, the value of the target variable is not dependent on the information for which the task was specified (which is often the case in other meta-learning applications^39^). The expected advantages of MAML over GNNs are in the adaptation phase. Once a new solute emerges, the GNN model would require full retraining, while MAML offers fast adaptation. The MAML algorithm was specifically created to allow fine-tuning from small sets in contrast to traditional GNNs.

There are two distinctions that have to be made, however. First, the model should be able to predict activity coefficients for systems with novel ILs. This issue has been already addressed in the literature.^27^ However, the model’s ability to operate with novel solutes remained unanswered. In this work, it is proposed to handle it with respect to meta-learning framework, i.e., with systems grouped into task with respect to the solute molecule. This formulation is simple, but it could be argued that there is a chemical rationale behind it. The model trained on data regarding some sequence of solute compounds (e.g., propane, butane, pentane) should be able to quickly adapt to the next element in that sequence (e.g., hexane). The activity coefficient value is dependent upon the chemical structures of the solvent and the solute. Consequently, for a common solvent and different solutes, some solvent–solute interactions would be shared between different solvent–solute pairs.

The used task definition has mostly a practical value, however. This framework tries to build a model that would help answer the question of which solvent is best suited for a particular solute. Even though more parameters are relevant for an adapted model’s performance (e.g., chemical space covered within a task, temperature range, measurement uncertainty, etc.), they are often unknown. In this utilitarian perspective, the proposed adapted models would be utilized to find the best solvent from limited data sets. Resolving the mentioned issues is relevant, but challenging upfront during the data collection. Our goal was to prepare a task definition that is ready to be easily used by the end users who might not be aware of the chemical space they will explore or test set they will select for validation. Consequently, a more complex task definition would hinder its usability by requiring the collection of more information regarding the system before modeling. It should be acknowledged, however, that the above-mentioned issues are potentially impacting.

The modeling framework, described verbally in the preceding text, is further summarized visually in Figure 2.

**Figure 2:**
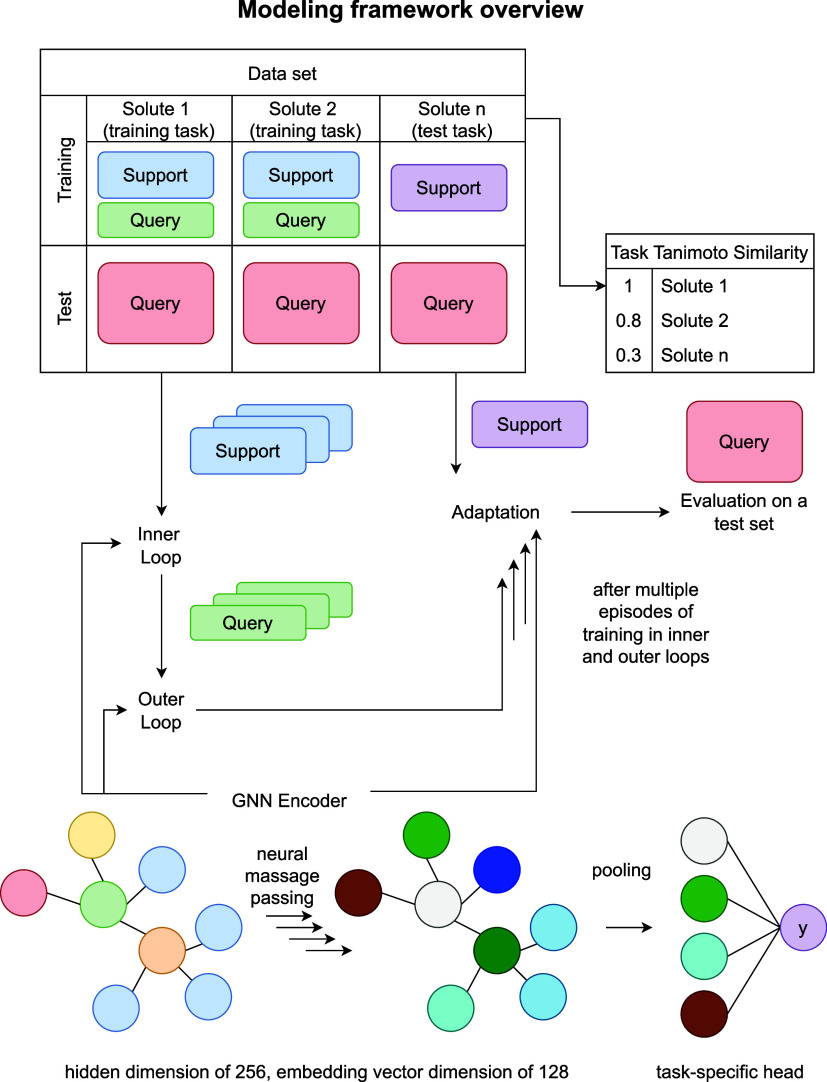
Visual explanation of the modeling framework.

## Results and Discussion

### Overview of the Data Set and Tasks’ Similarity

In this study, the data set on the infinite-dilution activity coefficients of various solutes in ILs was used. The data set comprises over 39,000 data points, spanning 109 solutes and 214 ILs (combinations of 94 cations and 38 anions).^27^ Data on the solubility of each solute in various ILs form a task.

Figure 3 illustrates the distribution of data points across the data set’s tasks. It is evident that there are comparable numbers of tasks with high (>256) and low (<100) data point counts. The graph shows that the prevalent issue of limited data availability across most tasks does not apply to this data set. In other literature, it was observed that this dominance of low-data tasks restricts the application of other approaches beyond meta-learning.^40^ However, since this issue is not as pronounced in this data set, it suggests that a comparison between MAML and GNN should be conclusive, as GNN has been demonstrated to yield reliable models when trained on this data set.^27^ Approximately 36 tasks have fewer than 100 data points available. These tasks often relate to less-studied systems that may hold scientific interest but are not ideal for testing modeling approaches. Conversely, approximately 20 tasks have exceptionally high data availability (of 800 or more).

**Figure 3:**
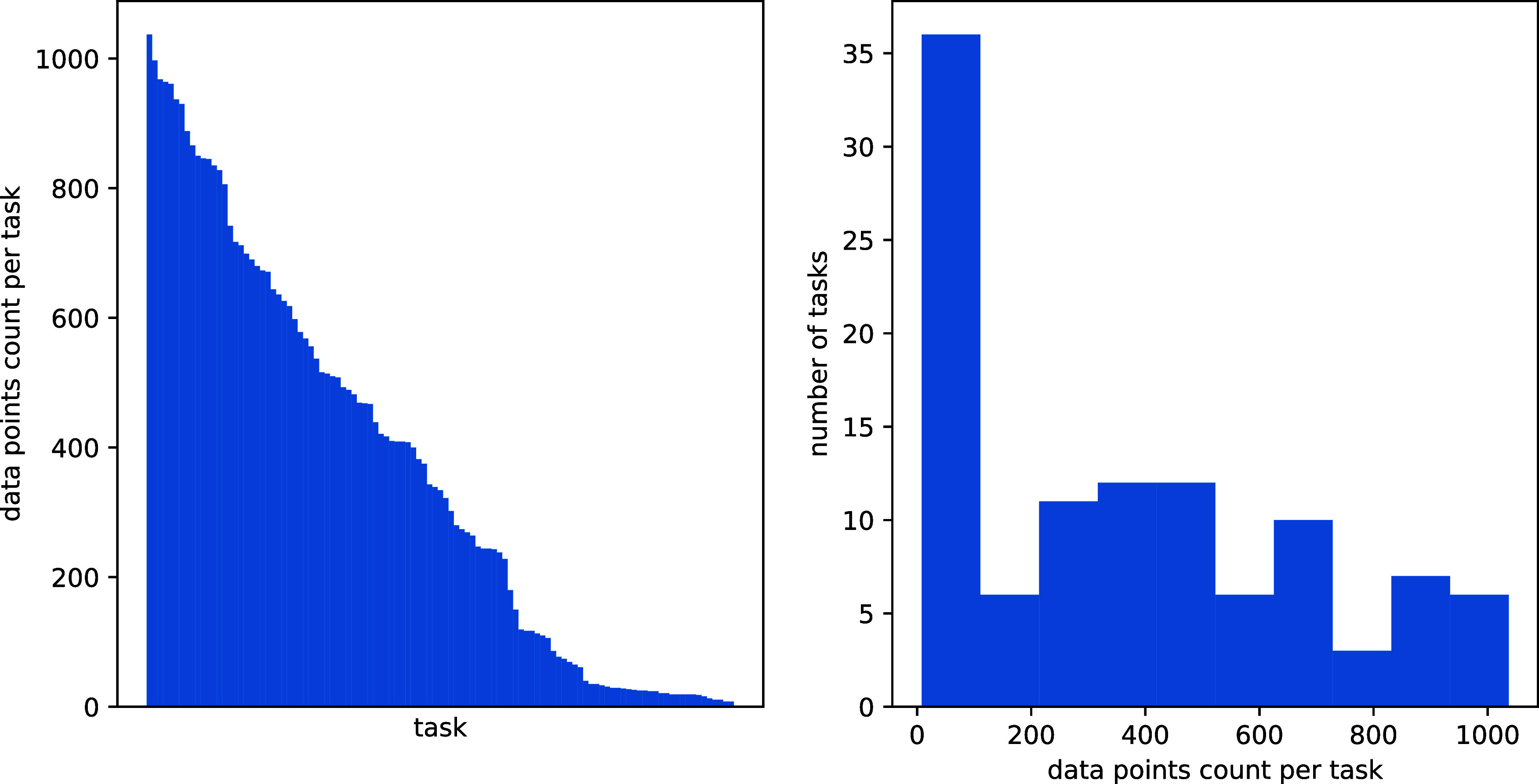
Data points count per task.

Figure 4 illustrates the pairwise calculated Tanimoto similarity among solutes (tasks) in the data set. The graph exhibits symmetry with the diagonal representing the calculation for the same compound, yielding a perfect similarity value of 1. It is evident that certain solutes exhibit a significant similarity to each other. One cluster corresponds to alkanes such as *n*-pentane, *n*-nonane, and *n*-decane, along with their analogues. It is hypothesized that a model trained using these compounds should be relatively straightforward to fine-tune for the remaining compounds. To some extent, a similar situation may occur for compounds sharing similar functional groups, such as between butanone and cyclopentanone. However, some compounds, like diisopropyl ether, are dissimilar to most of the other tasks but exhibit partial similarity to a few compounds that are ethers. Finally, there are compounds such as trichloromethane that are dissimilar to most of the compounds in the data set. It would be intriguing to assess whether adaptation to these types of compounds poses greater challenges for the MAML-based model, leading to poorer few-shot-learning performance.

**Figure 4:**
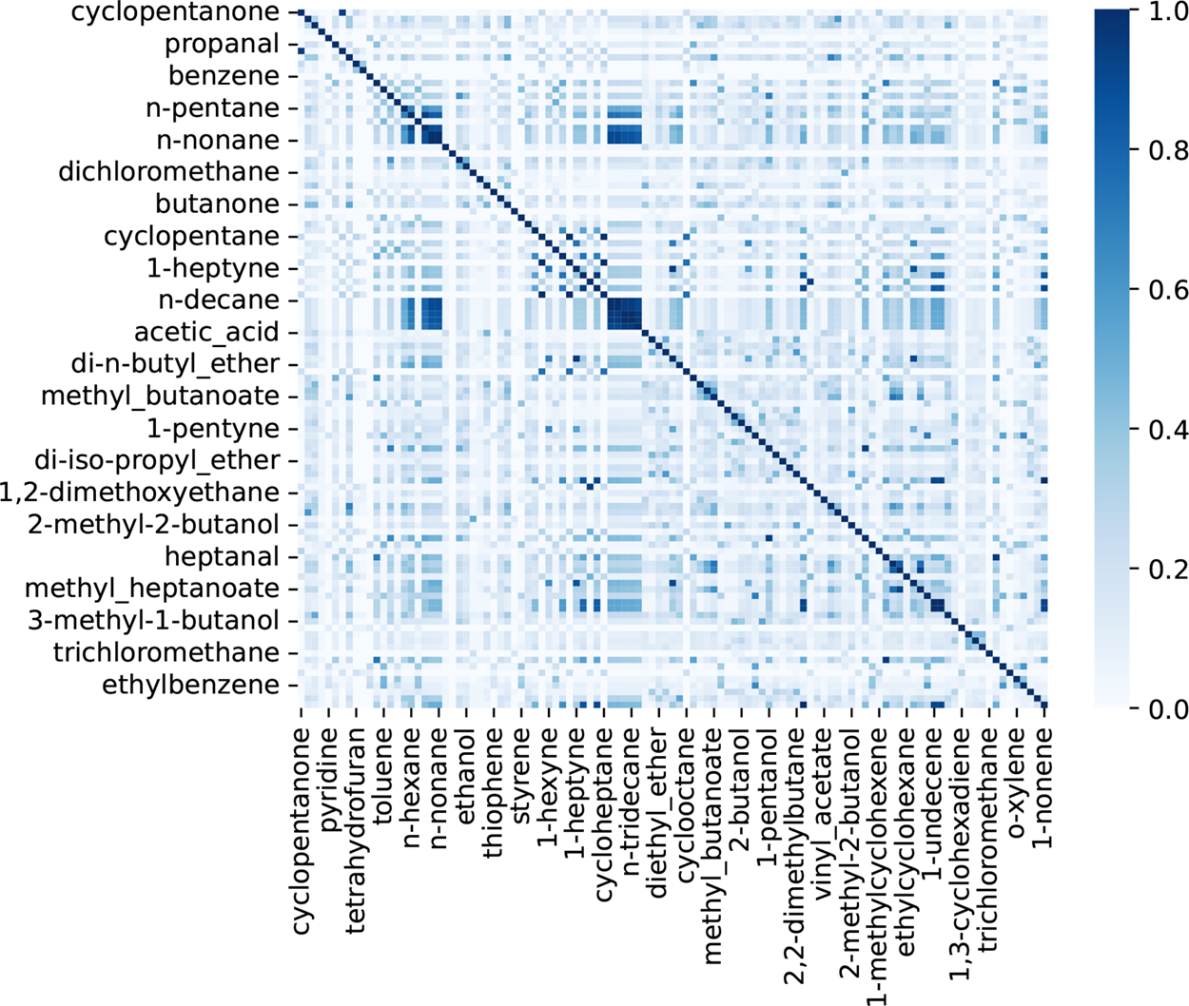
Tasks’ similarity as estimated by Tanimoto score.

Following the discussion above, it was decided to select a subset of closely similar tasks and another subset of dissimilar tasks to test how the MAML model adapts to new tasks. The illustration of chemical space across the randomly selected test tasks is shown in Appendix B (Supporting Information). The procedure of preparing those graphs followed the method described in our previous work, consequently utilizing molecular fingerprints (ECFP) reduced to 2D plots using the T-SNE algorithm and the Jaccard metric.^41^ Shortly, compounds similar in chemical space described with ECFP remain geometrically close to each other after reducing the space with T-SNE to two dimensions (referenced as Dim_1 and Dim_2 in Appendix B (Supporting Information). Those dimensions were calculated to mimic grouping patterns occurring in original multidimensional space. The information on temperature dependency was reduced to the inclusion of number of records for a particular IL–solute system (denoted with varying size of either gray or orange dots in Appendix B (Supporting Information) rather than including temperature across features for T-SNE fitting. The inspection of chemical space for highlighted tasks reveals that there is no evidence of significant biases in the test tasks that would render them unrepresentative of one another or of the overall task distribution.

### MAML Comparison to GNN on All Tasks

The modeling efforts commenced with a GNN model trained using conventional procedures. The GNN architecture was built upon our prior research,^9^ with modifications that incorporate additional graph convolutions for the solute molecule representation. The GNN was trained for 300 epochs, spanning batches that encompassed all tasks contributing to weight updates akin to traditional neural network training. Employing random search, the optimal convolution function was identified as the atom message passing-style protocol, as detailed in the Methods section.

Table 1 presents a comparative analysis of the GNN and MAML models. Notably, both models employed the same architectural framework. The distinction lies in the weight update protocol used by MAML, which employs training in batches of 32 tasks per outer loop. It is also worth noting that MAML used a smaller training set, with some data reserved for future adaptation (as described later). While it is evident that the MAML protocol resulted in an inferior out-of-the-box model performance, the primary objective remains to develop a model that can be readily adapted for specific tasks. Consequently, achieving competitive out-of-the-box performance for all tasks is not a prerequisite. However, it is worth acknowledging that the MAML training method was observed to require greater computational resources than DL approaches, in line with previous research (around 12 GPU h on NVIDIA A100 compared to around 20 min for classical GNN).^14^

**Table 1:** Comparison in Performance between Traditionally Trained GNN and GNN-Based MAML Models

test set metric	GNN	MAML
*R* ^2^	0.9304	0.6931
RMSE	0.3999	0.8398
MAE	0.2820	0.6122

Notably, the GNN model exhibited performance comparable to that reported in the literature.^27^ While the highest performance of DL models was reported using bootstrap and model ensemble, which significantly increased the computational cost of the method, the primary objective of this work is to investigate the applicability of MAML in the domain under investigation. Although the utilization of ensemble methods is not indispensable for achieving this goal, it is worth noting that this approach should be employed to optimize the model performance in subsequent studies and applications. The matching performance of single DL models facilitates a reliable comparison between the MAML model and the best-performing DL methods.

### MAML Comparison to GNN on Test Tasks

To study how MAML adapts to novel tasks, it is essential to select tasks for adaptation. To achieve this goal, we randomly selected 6 dissimilar and 6 similar tasks from the meta-training set, resulting in 12 test tasks out of 109 in the full data set.

Table 2 presents a comparative analysis of model performance on previously reserved test tasks. The classical GNN model was trained on the entire training data, and metrics were calculated separately for each task’s test set. In contrast, MAML modeling employed a distinct protocol: a meta-trained model was adapted separately for each task over a period of 30 epochs, with early stopping based on a validation subset composed of diverse ILs compared to the adaptation set. This targeted approach aims to facilitate the acquisition of reliable predictions from a limited number of examples. For this comparison, 128 samples per task were randomly selected.

**Table 2:** Comparison in Performance between a Traditionally Trained GNN and a GNN-Based MAML Model on Test Tasks

test compound (test task)	average RMSE on test task after adaptation 128-shot MAML	RMSE on test task classical GNN	similarity of the molecule to meta-training examples	number of classical training samples
*n*-octane	0.5257	0.5051	1.0	833
cyclopentane	0.3694	1.0003	1.0	430
cycloheptane	0.7316	0.7344	1.0	546
1-octyne	0.3267	0.3734	0.94	490
1-pentanol	0.6569	0.2671	0.93	204
1-hexyne	0.2486	0.2342	0.73	618
2-propanol	1.0399	0.3621	0.46	516
*tert*-butylethyl ether	0.4848	0.4344	0.44	227
diethyl ether	0.6345	0.6770	0.34	453
methanol	1.0455	0.4478	0.28	819
water	1.4426	0.6061	0.17	424
acetonitrile	0.2999	0.2184	0.17	536

As shown in Table 2, the MAML model outperformed the GNN model on certain tasks, even with only a subset of the data. This conclusion was observed for cyclopentane, cycloheptane, 1-octyne, and diethyl ether. In cases such as *n*-octane and 1-hexyne, performance was marginally inferior to that of the GNN model, even though the GNN was trained with a significantly larger number of data points. This is a notable achievement, highlighting MAML’s ability to learn representations that remain effective under limited-data conditions—an issue especially relevant to chemical property prediction.

However, it is important to note that the number of data points alone does not fully encapsulate the intricacies of the results. It is evident that, for the majority of test tasks that were similar to training tasks (five of six), the MAML model achieved comparable or even superior performance to the GNN model. Conversely, opposite conclusions were drawn for tasks that differed from training tasks, specifically for five of the six tasks. This underscores the crucial role of similarity between a test task and the meta-training tasks in determining the model’s predictive power.

Meta-learning framing of this problem seems favorable from a chemical standpoint as well. The neural network in meta-training might capture properties that are universally applicable across a task collection. In terms of modeling solvent–solute systems, these include fundamentals of thermodynamics and intermolecular interactions (electrostatic, hydrogen bonding, and π–π stacking). During the adaptation, nuances for a particular solute are learned by the model. This closely mirrors how chemists classically thought about modeling observed behaviors as a sum of general laws and specific molecular features.

Consequently, chemical similarity naturally guides this discussion into the problem of related tasks. For similar solutes, similar interaction patterns should occur. Knowledge should therefore be easily transferable to similar tasks. If the model was meta-trained on benzene sorption, it should be able to utilize the knowledge by analogy to predict systems with, e.g., toluene or xylenes, with very small data sets needed to adapt the model for their particular interactions with solvents.

The results indicate that the similarity of tasks affects the performance of the MAML. However, the analysis using statistical tools is constrained by the number of test tasks investigated. The 12 selected test tasks constitute approximately 11% of the tasks in the data set, which is a reasonable proportion.^9^ Further enlargement of this set might result in an inadequately small training set. Consequently, a procedure akin to cross-validation by the tasks was implemented.

Initially, test tasks were randomly chosen from 20 top similar and 20 top dissimilar tasks within the data set, ensuring a sufficient number of training instances and representation in the global test set. It is worth noting that the dissimilar tasks are exhibited rarely in the data set as depicted in Appendix C (Supporting Information). For the task cross-validation protocol, a similar approach was employed. First, 18 of 20 tasks in each group were randomly selected and divided into 3 folds. Each training-testing run comprised 6 similar and 6 dissimilar testing tasks, amounting to 18 tasks per group after 3 folds. For each task, the difference between the MAML RMSE metric value and the traditional GNN RMSE metric value was calculated. To mitigate the impact of outliers on the analysis,^27^ the difference in metrics was normalized (per task) by the GNN RMSE metric.

Given that the distribution of metric differences is not expected to follow a normal distribution—as evidenced by outliers in Table 2 for tasks involving cyclopentane, 1-pentanol, 2-propanol, methanol, and water (5 of 12 tasks)—the nonparametric Kruskal–Wallis test was employed. This test revealed that the median of relative difference between the two models is dependent on whether the task was similar or dissimilar, with a statistically significant *p*-value of 0.039 (*p* < 0.05).

### Tasks Similarity Importance for MAML Modeling

All performance metrics for the adapted MAML models are presented in Table 3.

**Table 3:** Performance Metrics of the GNN-Based MAML Model on Test Tasks after Adaptation on the 128-Shot Adaptation Set

test compound (test task)	average RMSE	average *R* ^2^	average MAE
*n*-octane	0.5257	0.8315	0.3832
cyclopentane	0.3694	0.7561	0.2600
cycloheptane	0.7316	0.5963	0.5785
1-octyne	0.3267	0.7876	0.2569
1-pentanol	0.6569	–0.5771	0.6038
1-hexyne	0.2486	0.8175	0.2079
2-propanol	1.0399	–1.6314	0.8014
*tert*-butylethyl ether	0.4848	0.1677	0.4648
diethyl ether	0.6345	–0.7834	0.3621
methanol	1.0455	–0.6377	0.6970
water	1.4426	–1.7119	1.2335
acetonitrile	0.2999	0.1668	0.2073

Although the metrics for tasks distinct from training tasks exhibit relatively small absolute error, the MAE metric values for these tasks remain relatively low. This suggests that the MAML model frequently proposes qualitatively accurate estimations (within an order of magnitude). Due to data scarcity and instability with respect to adaptation set selection for some tasks the negative *R* ^2^ value was obtained, however. While quantitative modeling alone may not be sufficient, the model can still provide limited insights for experimental chemists in unique or specialized tasks. In particular, qualitative insights from model predictions may provide valuable guidance on which candidate compounds to prioritize for laboratory exploration first. Selecting the most probable compounds based on the model’s outputs could serve as a pragmatic starting point for experimental validation, thereby optimizing resource allocation and accelerating the discovery process.

The observation of MAML’s poor performance on dissimilar tasks is not surprising, however. Solutes significantly dissimilar from training solutes contain scaffolds and functional groups that might not have been introduced to the model anytime during meta-training. Consequently, the model is able to provide qualitative estimation based solely on general patterns on principles of sorption process. Learning novel interactions should require much more data.

### MAML Few-Shot Fine-Tuning and Adaptation Set Size Requirements

Figure 5 illustrates the evolution of two key metrics, namely, *R* ^2^ and RMSE, in response to variations in the adaptation set size for the four most effective fine-tuned models. It is important to note that these models were adapted for tasks similar to those used in the training. Since the models for tasks dissimilar from those in the training tasks provided only a qualitative approximation of the target variable, studying their metrics in relation to the adaptation set size would not be informative. Appendix D (Supporting Information) provides the standard deviation of the metric values on top of the metric evolution.

**Figure 5:**
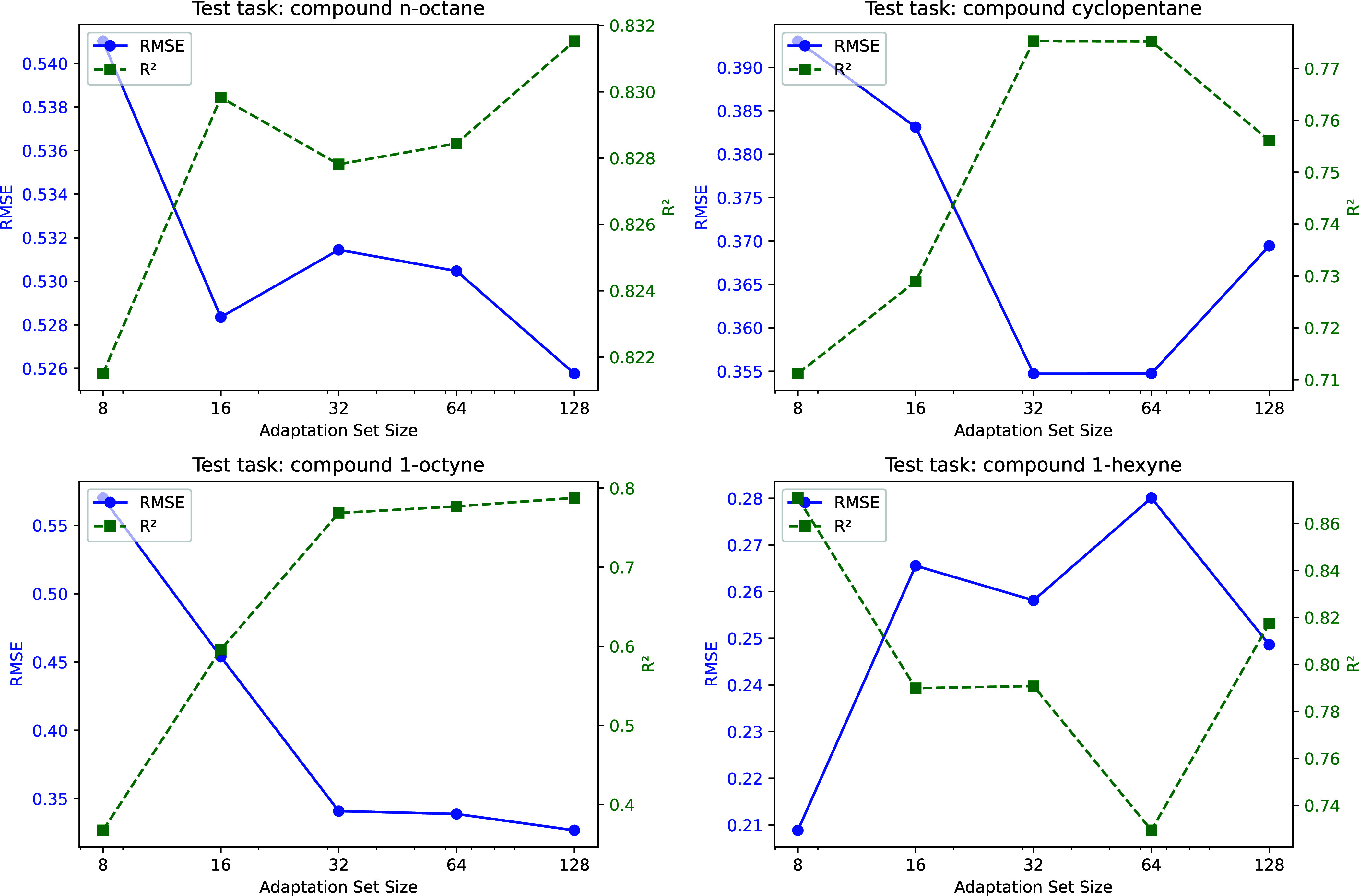
Change of test set metrics with adaptation set size.

It is evident that MAML demonstrated consistent performance even when fine-tuned on a limited number of adaptation samples. For *n*-octane, *R* ^2^ increased from 0.822 to 0.832 as the adaptation set size increased from 8 to 128 samples. It is plausible that the initial model weights already contained sufficient information for a reliable prediction.

However, this observation does not apply to models predicting 1-octyne IDAC in ILs. During adaptation, a model with an *R* ^2^ value of 0.35 was obtained using 8 samples. This metric was subsequently enhanced to 0.79 with 128 adaptation samples. This significant change in the metric value suggests that the model genuinely learned novel relationships that were not detected during meta-training. Given that alkynes were not predominantly present in the data set, it would be intriguing to assess whether this improvement in the metric resulted in an enhanced generalization across other tasks. It is possible that the model updated its weights to accommodate interactions with alkynes, thereby improving not only the performance for this specific task but also for other tasks. This issue was further explored in subsequent experiments.

Surprisingly, alterations in metrics were observed for cyclopentane and 1-hexyne. These changes may be attributed to the random selection of potentially nonrepresentative adaptation sets. This hypothesis is supported by comparing the metric values’ standard deviations. The reported metrics are averages over five different evaluations on the query set (drawn for each task from the test subset of the data set), while the adaptation set is randomly selected from the training subset. For example, for 1-hexyne, the *R* ^2^ value with an adaptation set size of 64 is *R* ^2^ = 0.73 ± 0.28, while for 128 samples, it is *R* ^2^ = 0.818 ± 0.072. As a result, the difference is not statistically significant. Similar conclusions are drawn when examining the metrics for the cyclopentane model. Both tasks were fine-tuned using relatively small subsets of the training instances: up to 128 of 430 and up to 128 of 618, respectively. It is plausible that a more effective strategy for selecting adaptation sets could be employed to reduce variability and ultimately enhance model performance. This aspect is further investigated in subsequent experiments.

### Fine-Tuned MAML Models’ Versatility

Model fine-tuning involves adapting the model’s weights to novel tasks. This adaptation may result in modifications to a few weights or to a substantial portion of them. Consequently, the model after fine-tuning may exhibit diminished performance for other tasks compared to its initial performance. This phenomenon is occasionally referred to as catastrophic forgetting,^42^ as the model appears to forget the knowledge acquired during pretraining. Given that the two-step protocol—meta-training and adaptation—is crucial to MAML, it would be intriguing to assess the extent to which it affects MPP modeling.

The RMSE values computed on the complete test data set (which encompass all tasks) for MAML before and after adaptation are presented in Table 4.

**Table 4:** Performance Metric on the Full Test Set (for All Tasks) before and after Adaptation of the MAML Model

test compound (test task)	RMSE on full test set before adaptation	average RMSE on full test set after adaptation	relative percentage change	similarity of the molecule to meta-training examples
*n*-octane	0.8398	0.8464	–0.78	1.0
cyclopentane	0.8398	1.1827	–40.83	1.0
cycloheptane	0.8398	1.0568	–25.83	1.0
1-octyne	0.8398	0.8021	4.5	0.94
1-pentanol	0.8398	1.0385	–23.66	0.93
1-hexyne	0.8398	0.771	8.2	0.73
2-propanol	0.8398	1.0533	–25.42	0.46
*tert*-butylethyl ether	0.8398	0.9227	–9.88	0.44
diethyl ether	0.8398	1.7785	–111.77	0.34
methanol	0.8398	0.8462	–0.77	0.28
water	0.8398	1.8404	–119.14	0.17
acetonitrile	0.8398	0.8412	–0.16	0.17

As one might see, the model occasionally achieved comparable performance on a test set comprising all tasks before and after adaptation.

In exceptional instances, such as 1-octyne or 1-hexyne, even an enhancement in the model versatility was observed. This observation may be attributed to the fact that alkynes were generally less prevalent in the training set compared to alkanes, alcohols, or ketones. Consequently, their inclusion in the model could be more advantageous as it augments the model’s ability to generalize to diverse chemical functional groups.

However, it is not the case that model adaptation for tasks highly similar to the training tasks is associated with a modification of model weights that leaves the overall performance unaffected. For test tasks such as cyclopentane and cycloheptane, after adaptation, the overall performance, as measured by the RMSE, declined by 40 and 25%, respectively.

There is no discernible correlation between the relative percentage change of the metric value and task similarity. Given that fine-tuning is a multifaceted process governed by gradient-based optimization methods, it is unsurprising that no single factor predominantly influences its trajectory. Further research on continual learning is necessary to assess protocols that could effectively mitigate the risk associated with model fine-tuning.

From a chemical standpoint, it is expected that adaptation to dissimilar tasks would occasionally be related to diminished performance on other tasks. The neural network models encode chemical correlations in their weights. A less complex adaptation (e.g., to a solute that contains a slightly longer side chain) should, conceptually, require modification of only a few weights. For dissimilar compounds, novel interaction patterns might be encoded in place of weights that encode more principal rules governing the process.

However, it is worth noting that the well-known techniques like weight freezing or replay can be utilized to slightly decrease the negative impact of catastrophic forgetting, as evidenced in Appendix E (Supporting Information). One could limit this effect to 8 of 12 tasks and 6 of 6 dissimilar tasks by freezing selected (graph convolution) neural network layers. Similarly, replay usage reduces the RMSE of the full test set on 9 of 12 tasks and 4 of 6 dissimilar tasks. Likely, the high RMSE postadaptation in regular MAML arises because the model updates its weights to capture structural patterns relevant solely to novel, dissimilar tasks.

### Rational Selection of Adaptation Set

It might be argued that the issue of tasks’ similarity impact on meta-learning might be handled with just a proper adaptation set selection. The rationale for that hypothesis is that maybe the random selection of adaptation set is suboptimal for those tasks. If the model was adapted with solvents spanning across a wide chemical space, it might adapt more easily. In that context, the neural network might adapt its weights based on samples more evenly distributed in chemical space.

The inherent heterogeneity of chemical data poses a major challenge, arising from variations in the molecular systems and experimental conditions. In the context of few-shot learning, selecting adaptation sets is crucial for improving the model performance. One approach involves randomly selecting an adaptation set from the data set, while another method entails grouping the data points and selecting from the formed clusters. It was determined to cluster the data points from each test task, based on IL structure, into two, four, or eight groups, and to randomly select from each cluster until a total of 128 adaptation data points is obtained. Table 5 shows the impact of informed adaptation set selection on model performance across a range of test tasks, both similar and dissimilar to the training tasks.

**Table 5:** Performance Metrics of the GNN-Based MAML Model on Test Tasks after Adaptation on the 128-Shot Adaptation Set Selected Randomly, Using 2, 4, or 8 Clusters on IL Structures

test compound (test task)	average RMSE random selection	average RMSE 2 clusters selection	average RMSE 4 clusters selection	average RMSE 8 clusters selection
*n*-octane	0.5257	0.5468	0.5484	0.5629
cyclopentane	0.3694	0.5570	0.5930	0.6126
cycloheptane	0.7316	0.7806	0.7827	0.8129
1-octyne	0.3267	0.6149	0.5957	0.6079
1-pentanol	0.6569	0.6113	0.5528	0.7304
1-hexyne	0.2486	0.3465	0.2942	0.2474
2-propanol	1.0399	0.8748	0.8865	0.9068
*tert*-butylethyl ether	0.4848	0.6080	0.6329	0.6281
diethyl ether	0.6345	0.4312	0.4829	0.4766
methanol	1.0455	0.9350	1.0178	0.9644
water	1.4426	0.8259	0.8234	0.8128
acetonitrile	0.2999	0.3062	0.2800	0.3097

For test tasks that closely resemble the training tasks, the informed adaptation set selection yielded only a limited performance improvement. Specifically, it led to enhanced performance for only one task, 1-pentanol, while maintaining the same level of performance for 1-hexyne. This suggests that for tasks highly similar to the training data, the benefits of an informed selection may be minimal or nonexistent.

Conversely, for test tasks dissimilar from the training tasks, the informed adaptation set selection yielded more promising results, with improvements observed across five of six tasks. However, it is important to note that the performance in the random scenario was notably poor and the improvements achieved through the informed selection protocol were not substantial enough to significantly enhance the overall performance. Although the clustering-based selection of adaptation sets showed a slight increase in the reliability of fine-tuned models for dissimilar tasks, it did not elevate them to the level of well-performing quantitative models.

Overall, the practical significance of the rational selection of the adaptation sets appears to be limited. The findings suggest that while informed selection may offer some advantages, particularly for dissimilar tasks, it does not currently provide a substantial improvement in the model performance. Nevertheless, further experimental studies are warranted to explore potential strategies, akin to experimental design, that could optimize MAML performance under minimal data conditions. Such studies would be necessary to determine whether a more effective approach to adaptation set selection exists that could lead to superior model performance. Unfortunately, the current data set, which relies on randomly selected samples (regarding both the temperature and chemical space), does not permit such an analysis. Therefore, additional research is needed to address these gaps and develop more robust methods for selecting adaptation sets in machine-learning applications.

### Exploring Meta-Learning Targeted for Modeling Dissimilar Tasks

The MAML model limitations were apparent when adapting to task dissimilarity (as expressed by Tanimoto similarity between molecules representing tasks) from tasks used in meta-training. Consequently, it could be stated that these tasks were examples of the more challenging problem of out-of-distribution adaptation. In addition to the differing data distributions, the MAML algorithm itself also presented some limitations with respect to this issue. The MAML model performed worse before adaptation (Table 1). Furthermore, the MAML algorithm did not guide the optimization process to promote more-difficult tasks. Consequently, trials to improve the model for dissimilar tasks should address both of these issues. Obtaining an overall performance comparable to that of GNNs after solely meta-training may be achievable with a different meta-learning algorithm. The assumption was that the closer the meta-learning algorithm resembled the traditional GNN protocol, the higher the likelihood of achieving high metrics before the adaptation. This issue is important because no pretrained feature-extraction layers were utilized. Consequently, it is vital to ensure that convolution layers extract all of the important structure–property correlations.

On the basis of the above-stated rationale, the Reptile algorithm^39^ was selected. This method utilizes a stochastic gradient descent (SGD) optimizer in order to perform meta-learning in a less computationally expensive way than the MAML. The Reptile algorithm performs several (*k*) SGD steps on the task and then updates the initial parameters on the basis of the final parameters obtained at the end of these steps. If *k* is set to 1, the procedure closely resembles traditional GNN training but utilizes small batches containing just one task each. The change from MAML to Reptile and its derivatives required slight changes in model architecture, however. The batch normalization used in our previous work and the MAML model were unstable during Reptile training. Its impact was probably limited in MAML, since MAML utilized in meta-training two sets (a support set to update the internal model and a query set to calculate the loss). Omitting batch normalization did not significantly affect overall metrics for the MAML model. As Reptile used a simpler meta-learning loop, batch normalization on very small few-shot batches had a more significant impact on the training. Furthermore, graph convolutional layers were not updated during adaptation, since the Reptile model was expected to yield better overall performance before adaptation. The comparison between GNN, MAML, and Reptile variant utilizing all common architectures is provided in Appendix F (Supporting Information).

Adjusting the Reptile algorithm for out-of-distribution task targeting required changes to the training procedures. It was decided to use a simple approach of scaling the loss function by weights proportional to the similarity among the tasks. This method is termed Task Similarity-Aware Reptile (TSA-Reptile). The rationale for the proposed method is that out-of-distribution tasks should be given higher priority as they are more challenging to adapt to. The Reptile algorithm can be visualized geometrically as aiming to reach the average of the loss minima across the tasks. Since tasks similar to training tasks can be easily solved (e.g., via traditional MAML, as shown above), TSA-Reptile could focus on obtaining a model closer to minima across more challenging tasks. This was achieved by scaling the loss based on the Tanimoto task similarity. The scaling was needed during meta-training rather than during adaptation to obtain an initialization that could be easily fine-tuned across both similar and dissimilar tasks. The three main approaches to implementing TSA-Reptile were tested: linear, exponential, and inverted. All the scaling factors were proportional to factor *S*, which represents the Tanimoto similarity of the task to the most similar example in the meta-training set. In the linear approach, the scaling factor was of the form 2 – S. This approach resulted in assigning regular loss to samples from tasks resembling other meta-training tasks, as the scaling factor approaches 1 when *S* approaches 1. On the contrary, the scaling factor is limited to 2 if the task is fully dissimilar from the training tasks. Consequently, the scaling factor changes linearly with *S*. On the contrary, the exponential and inverted methods use functions of the form exp­(−*S*) and 1/*S*, respectively. It should be acknowledged that changing both architecture and algorithm at the same time perplexes analysis. The architectural changes that benefited Reptile also, to some extent, benefit the MAML. The analysis that utilizes all models with a common architecture is provided in Appendix G (Supporting Information).

Table 6 shows a comparison among MAML, Reptile, and TSA-Reptile. It is clear that the results were highly dependent on task similarity. For tasks similar to meta-training tasks, MAML outperformed other methods. In contrast, TSA-Reptile significantly outperformed the MAML method on dissimilar tasks. Among the three tested TSA-Reptile models, the linear scaling performed best. The TSA-Reptile with linear scaling achieved the best performance on 5 of 6 tasks with low similarity to the training tasks. This observation could not be attributed to just modifications of the architecture, however. The TSA-Reptile outperformed MAML with Reptile architecture on 4 of 6 dissimilar tasks, as evidenced in Appendix G (Supporting Information).

**Table 6:** Performance Metric of MAML, Reptile, and TSA-Reptile on Test Tasks^a^

test compound (test task)	average RMSE (MAML model)	average RMSE (reptile model)	average RMSE (TSA-reptile with linear scaling model)	similarity of the molecule to meta-training examples
*n*-octane	**0.5257**	0.6545	0.6404	1.0
cyclopentane	**0.3694**	0.6163	0.6500	1.0
cycloheptane	**0.7316**	0.8197	0.8574	1.0
1-octyne	**0.3267**	0.5867	0.6043	0.94
1-pentanol	0.6569	0.3840	0.5801	0.93
1-hexyne	**0.2486**	0.3637	0.3767	0.73
2-propanol	1.0399	0.4152	**0.4115**	0.46
*tert*-butylethyl ether	0.4848	0.4888	**0.3618**	0.44
diethyl ether	**0.6345**	0.8510	0.8449	0.34
methanol	1.0455	0.5869	**0.5033**	0.28
water	1.4426	0.5398	**0.4812**	0.17
acetonitrile	0.2999	0.1593	**0.1496**	0.17
full test before f.t.	0.8398	0.6707	**0.6564**	-

a The best RMSE value for each task is shown in bold font.

The TSA-Reptile model allows for providing quantitative models even for the most challenging tasks. The models adapted for the three most dissimilar test tasks—methanol, water, and acetonitrile—obtained *R* ^2^ metrics of 0.62, 0.70, and 0.79, respectively. The TSA-Reptile method was shown to be a better choice for out-of-distribution task adaptation. It did not outperform MAML for all the test tasks, however. The proper selection of the algorithm between TSA-Reptile and MAML is dependent on the users’ ultimate goal in using the prediction models.

Table 7 shows comparison of different TSA-Reptile variants. Along with the RMSE metrics, the average ranks of each model are provided. The rank represents the average of the model’s ranks across all benchmarking tasks, with 1 being the best and 5 being the worst. It is worth noting that the application of exponential and inverse scaling methods can yield weight values that are either excessively high or low. Specifically, in the first method, which employs an exponential function, the weights range from approximately 0.3679 to 1. In contrast, the second method, utilizing an inverse scaling approach, produces weights ranging from approximately 1 to 10^10^. These ranges differ substantially from the linear scaling option, which spans between 1 and 2. To mitigate the risk of numerical instability in optimization algorithms, it is crucial to prevent weights from becoming overly large or small. Therefore, normalization of the weights is applied in these scenarios, wherein each calculated weight is divided by the sum of all weight values. This normalization process ensures that the weights can be interpreted as probabilities, thereby preventing any single task from dominating the learning process and promoting a more equitable influence across all tasks. Furthermore, normalized weights facilitate a more balanced comparison between different tasks. Even though the theoretical discussion on weights assigned to tasks seems relevant, the experimental validation showed a lower importance of those risks. Consequently, the normalization of weights was more impactful for the adapted models’ performance than the used scaling function. The inverse scaling function application allowed to even further reduce the error on dissimilar tasks. It was often associated with even higher errors on similar tasks than in the linear variant of TSA-Reptile.

**Table 7:** Performance Metric of TSA-Reptile Variants on Test Tasks with Their Average Rank on the Tasks^a^

test compound (test task)	average RMSE (TSA-Reptile with linear scaling model)	average RMSE (TSA-Reptile with linear scaling model and weights normalization)	average RMSE (TSA-Reptile with exp. scaling model)	average RMSE (TSA-Reptile with exp. scaling model and weights normalization)	average RMSE (TSA-Reptile with inverse scaling model)	average RMSE (TSA-Reptile with inverse scaling model and weights normalization)	average RMSE (MAML model)
*n*-octane	0.6404	1.0437	**0.5117**	1.0397	0.6899	1.0383	0.5257
cyclopentane	0.6500	0.8759	0.5123	0.8765	0.6544	0.8039	**0.3694**
cycloheptane	0.8574	0.8472	0.8000	0.8322	0.8194	0.8038	**0.7316**
1-octyne	0.6043	0.9510	0.5061	0.9348	0.6323	0.8978	**0.3267**
1-pentanol	0.5801	**0.2873**	0.3889	0.2896	0.3728	0.3096	0.6569
1-hexyne	0.3767	0.6933	0.3514	0.7158	0.4159	0.6814	**0.2486**
2-propanol	0.4115	0.6778	**0.2982**	0.6763	0.3385	0.6857	1.0399
*tert*-butylethyl ether	0.3618	0.6759	0.3911	0.6795	**0.3091**	0.6313	0.4848
diethyl ether	0.8449	0.8536	0.8266	0.8634	0.8417	0.8825	**0.6345**
methanol	0.5033	0.7799	**0.4243**	0.7725	0.4811	0.7778	1.0455
water	0.4812	0.7395	0.5715	0.7280	**0.4240**	0.7323	1.4426
acetonitrile	0.1496	0.1656	0.1635	0.1604	**0.1366**	0.1614	0.2999
full test before f.t.	0.6564	0.9591	**0.5117**	0.9488	0.6262	0.9358	0.8398
average rank	3.42	5.58	2.42	5.08	2.83	4.83	3.83
average rank (dissimilar tasks only)	2.67	5.67	2.5	4.67	1.67	5.33	5.5

a The best RMSE value for each task is shown in bold font.

The added value of meta-learning most probably stems from learning objective to “learn how to learn”. The performance of the meta-learning models is not expected to be solely a consequence of utilizing the knowledge gained during meta-training, as depicted by comparison in Appendix G (Supporting Information). Consequently, meta-learning seems to be an interesting approach toward few-shot learning in the context of sorption with ILs, their predominant application area.

### Modeling Limitations

The main contribution of this study is to discuss tasks’ similarity impact on meta-learning modeling. While this concept is grounded in chemical intuition, its undisputed quantitative estimation is challenging. In this work, we utilized Tanimoto similarity as it is a simple and commonly used metric. The utilization of other metrics of compounds’ similarity might be interesting. The utilization of an average similarity to meta-training tasks (rather than top-1 value in this study) might also allow the study of this problem from a different angle. Nevertheless, the issue of task similarity is expected (from the perspective of both chemistry and computer science) to impact the adapted models’ performance.

The availability of data for meta-training remains a significant limiting factor across various research endeavors. In studies focused on small organic compounds, researchers have successfully utilized large data sets from the ChEMBL database.^14^ However, to the best of our current knowledge, there are no comparable solutions (on a scale similar to that of ChEMBL) in the domain of modeling IL–solute systems. This lack of extensive data resources poses a challenge to advancing research in this area.

The nature of MAML research restricts the feasibility of studying multiple data sets within a single study. Given that the objective is to leverage pretraining on extensive tasks, most similar problems are frequently consolidated into a single data set. In contrast, the literature on MAML modeling for sorption, particularly for designer solvents such as ILs, is comparatively scarce compared with studies focused on small organic compounds.

The application of extensive data sets, which are typically suitable for small organic molecules, is constrained when examining solvents, such as ionic liquids. Previous research has unequivocally demonstrated that solution modeling frameworks, commonly developed for conventional organic compounds, often encounter limitations and fail when directly applied to ILs.^43^

The selection of ILs for this study was influenced, to some extent, by data availability. Although data on solvents other than ILs are available, they are often provided at a significantly lower scale than for ILs^44^ or are housed in paid repositories.^45^ This limitation reduces their utility in the scientific research endeavors.

While this study focuses on modeling solvent–solute systems, ML application for IL research is more perplexing. The proposed TSA-Reptile method is not well-defined outside of sorption settings, where task similarity can be unambiguously defined with Tanimoto similarity. TSA-Reptile could further enhance its predictive performance, as it was shown to outperform both MAML and GNN on dissimilar tasks. It would not be a simple replacement in the architecture; for in-distribution tasks, MAML is a preferred choice. At this point, comparison is limited by the utilization of different benchmarks, which are limited to sorption-like tasks. However, it must be mentioned that there are emerging and easier data collection possibilities based on agentic LLMs.^46^ In those multiproperty settings,^46^ the possibility of incorporation of meta-learning principles, including MAML and TSA-Reptile, will be explored in the future.

Assessing the impact of data quality on MAML modeling could be a valuable area of exploration akin to the methodology used in our earlier study. However, it is important to note that the potential anomalies identified in our previous research concerning the physicochemical properties of ILs are not relevant in this context. In a few-shot learning scenario, data quality remains a crucial factor, and it is anticipated that a superior data quality will lead to more effective models. We are confident that using a data set previously extracted and meticulously cleaned in the literature^27^ will provide an acceptable level of data quality for this research endeavor.

Further research is needed to assess the applicability of the MAML framework for sorption modeling, particularly when using various target variables such as mole or mass fractions of solutes in ILs. This modeling endeavor would necessitate the acquisition of a comprehensive and distinct data set. At present, the literature primarily provides data sets focused on limited task selections, such as gas^47^ or active pharmaceutical ingredients.^48^ However, the IDAC data set appears to offer the largest number of tasks and the greatest variability. Given that the primary objective of this study was to evaluate the performance of the MAML method in modeling IL–solute systems, it was advantageous to analyze a well-established benchmark with the highest available number of tasks. Both of these criteria are best met by the IDAC data set used in this study.

Although the limitations mentioned above exist, they do not preclude the study’s primary observations on task similarity, adaptation set size requirements, and the impact of adaptation on models’ versatility.

## Summary

The recent challenge of low-data machine learning has been addressed by employing algorithms that perform meta-learning, i.e., they are trained to adapt even with limited data. Among these techniques, MAML presents a compelling solution to the data scarcity challenge in molecular property prediction. Several issues warrant further discussion before applying this method to the modeling of sorption phenomena. Its robustness and comparison to protocols based on joint training for multiple tasks need exploration. The correlation between metric enhancement and adaptation set size should be investigated. The impact of model adaptation on performance on other tasks warrants further research. Optimizing the model performance with an adaptation set from the training subset is intriguing. To validate this method, a large data set of activity coefficients for a wide range of solutes in ionic liquids was selected.

Although MAML’s predictive metrics often aligned closely with those of the GNN models, the important point is that MAML delivered this performance with markedly less data. This represents a meaningful advancement, demonstrating that meta-learning can mitigate the challenges posed by heterogeneous, sparsely available chemical data sets. Its limitation was observed when adapted to task that were out-of-distribution from meta-training tasks. The proposed TSA-Reptile model was shown to outperform MAML on adaptation to dissimilar tasks.

The findings emphasize the pivotal role of alignment between a test task and the tasks used in meta-training in gauging the model’s predictive capabilities. The study found that qualitative estimations in a few-shot learning manner are feasible for tasks dissimilar to training tasks. However, the practical significance of rationally selecting adaptation sets (regarding both temperature and chemical space) is limited. There is no clear correlation between task similarity to training tasks and the relative percentage change in the overall model’s performance on the full test set before and after adaptation. Further research on MAML is necessary, particularly in learning with a limited number of tasks during meta-training and in addressing catastrophic forgetting after fine-tuning.

## Supplementary Material



## Data Availability

The underlying code for this study is available and can be accessed via the link to the GitHub repository at: 10.5281/zenodo.19153451. The files containing requirements on freely available packages needed to reproduce the work are provided in the repository. Data sources are provided in the manuscript text.
