# Supplementary material for: Exploring Chemoinformatics Aspects of Few-Shot Meta-Learning by Example of an Infinite Dilution Activity Coefficient in Ionic Liquid Prediction
Source: J Chem Inf Model. 2026 Apr 13;66(8):4439–53. doi: 10.1021/acs.jcim.6c00067 (PMC13298820; doi:10.1021/acs.jcim.6c00067)
Supplement: Supplementary file 1 [file ci6c00067_si_001.pdf]

# Supporting Information: Exploring Chemoinformatics Aspects of Few-Shot Meta-Learning by Example of an Infinite Dilution Activity Coefficient in Ionic Liquid Prediction

Karol Baran* and Adam Kloskowski

*Department of Physical Chemistry, Faculty of Chemistry, Gdansk University of Technology, Narutowicza Str. 11/12, 80-233 Gdansk, Poland*

E-mail: karol.baran@pg.edu.pl

Phone: +48 58 347 25 93

## Contents

**Appendix A: Extended learning protocols details** **3**

**Appendix B: Test tasks' chemical space compared to chemical space of the full data set** **5**

**Appendix C: Histograms of tasks' similarity values as estimated by Tanimoto score** **6**

**Appendix D: Change of test set metrics (with associated standard deviation) with adaptation set size** **7**

**Appendix E: Performance metric on full test set (for all tasks) before and after adaptation the MAML model** 8

**Appendix F: Ablation study on architectural changes impact while transitioning from MAML to Reptile model** 9

**Appendix G: Comparison between MAML, Reptile, and fine-tuned GNN** 10

# Appendix A

## Extended learning protocols details

The Graph Neural Network model was built based on our previous research,[1] incorporating the same neural network architecture. To optimize the remaining hyperparameters, including the learning rate and the convolution function, random search was employed. Three primary convolution function types were evaluated: graph convolutional networks (GCN), graph attention networks (GAT), and Atom Message Passing style. The latter was reimplemented by integrating the original Chemprop[2] implementation into a PyTorch Geometric-compatible format, enabling the utilization of all functions within a unified environment. The learning rate values of 1e-3 and 5e-4 were tested. The models were developed utilizing the PyTorch[3] and PyTorch Geometric[4] Python libraries. The specifics regarding the employed methodology are detailed in our preceding research.[1] The details on model architecture, hyperparameters values and their search are available in our previous work and in the source code supporting this work.[1]

The MAML model utilizes different approach. In MAML, the loss function is optimized to update the model weights so that the model minimizes loss across all tasks. Consequently, a model serves as a compromise between separate models for each task, enabling it to adapt more swiftly to unseen tasks during testing. The initial step, known as the inner loop, focuses on task-specific adaptation. During this phase, the model is trained on a support set comprising data specific to a particular task. In this study, the support set included activity coefficient data for specific solutes (e.g., water or ethanol) across varying ILs. During the meta-training phase, the support set comprised 128 data points, while the remaining task data was utilized as the query set. The second step of the MAML protocol, referred as the outer loop, involves meta-parameter optimization. The errors identified during the evaluation of the query set are utilized to update the model's initial parameters. This iterative process is repeated across various tasks. The hyperparameters of MAML model were also optimized using a random search. The hyperparameters space included the inner learning rate (from

5e-5, 5e-4, 1e-4), outer learning rate (from 5e-6 and 5e-4), number of tasks contained in a batch task (from 2, 4, 32, 97 tasks), and the number of meta-training episodes (from 500, 10000, 60000). This approach was employed using a randomly selected validation subset drawn from the training subset.

# Appendix B

## Test tasks' chemical space compared to chemical space of the full data set

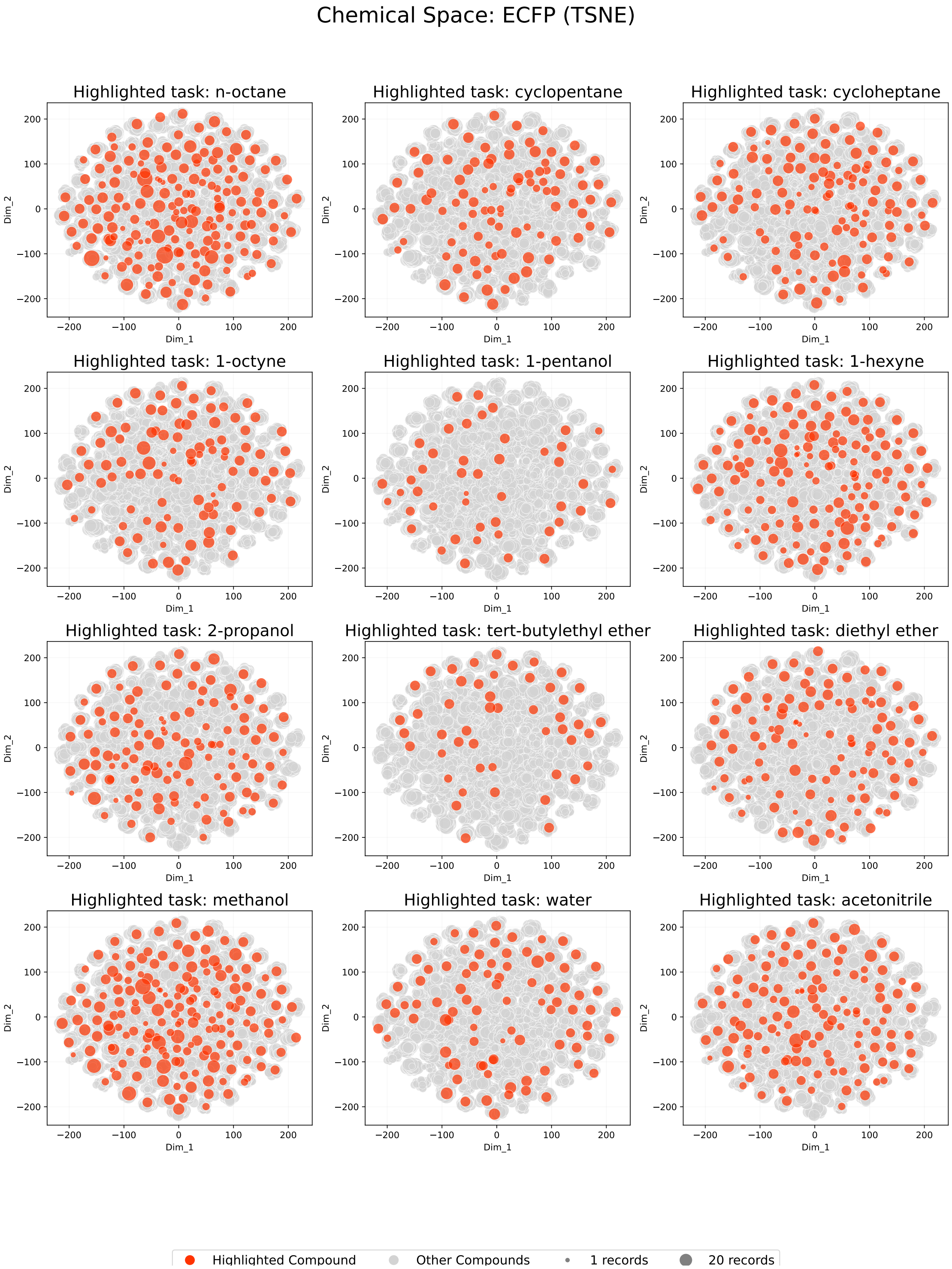

# Appendix C

## Histograms of tasks' similarity values as estimated by Tanimoto score

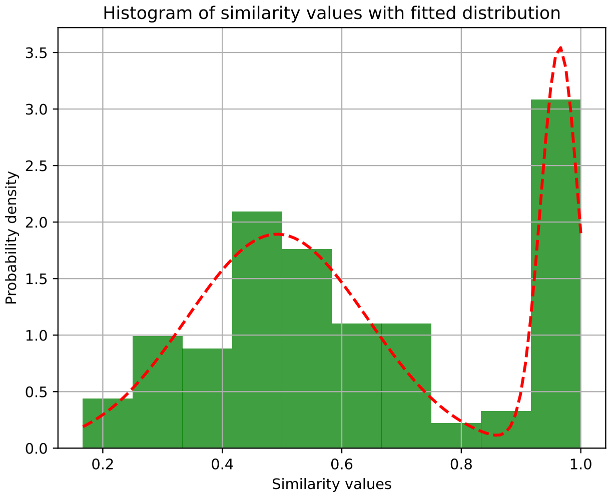


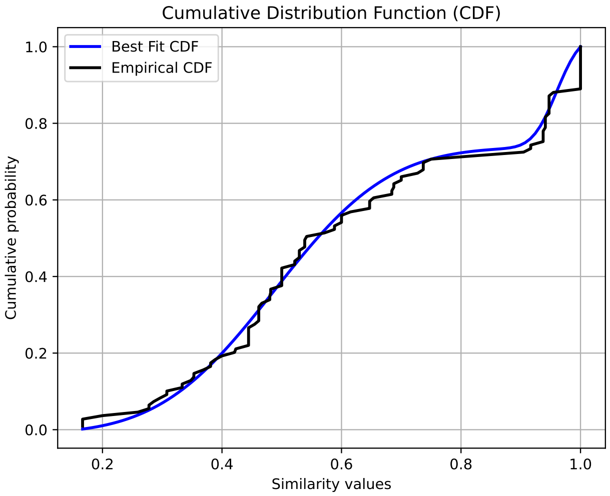

# Appendix D

## Change of test set metrics (with associated standard deviation) with adaptation set size

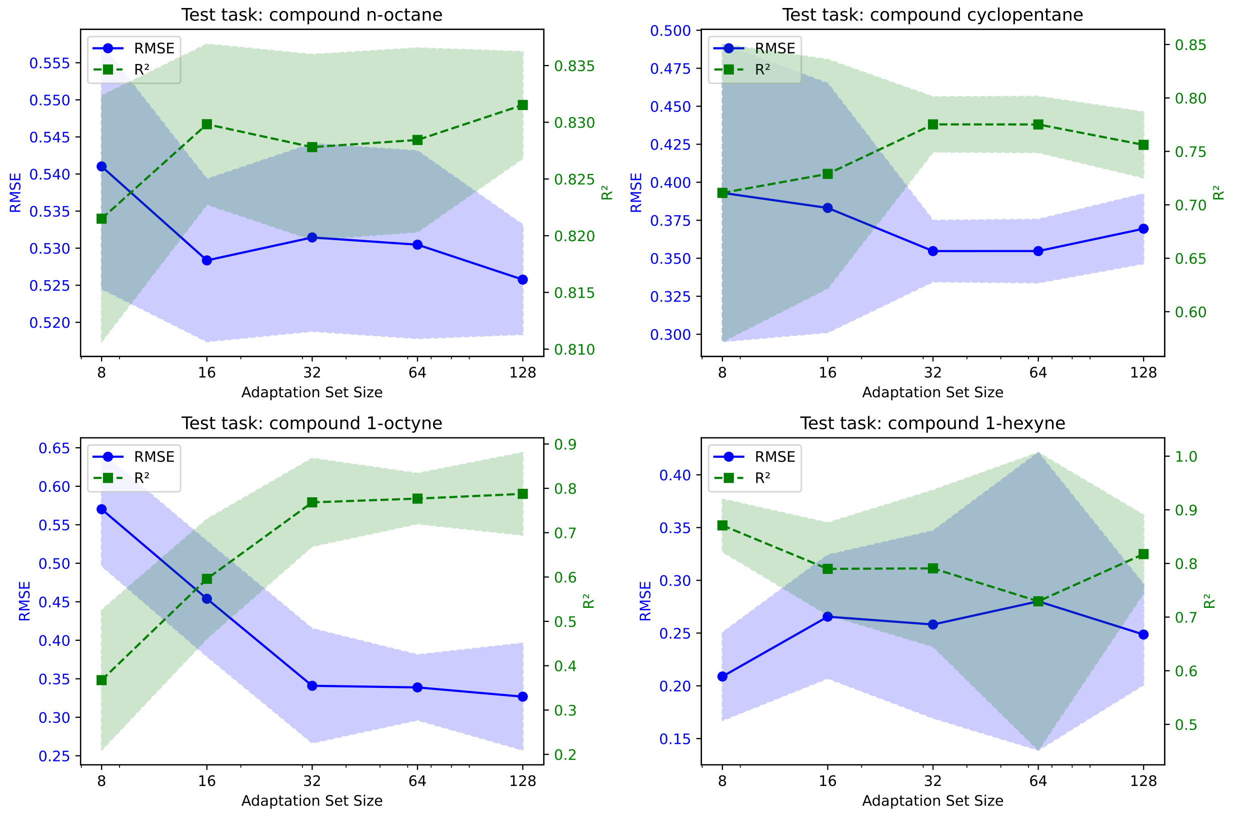

# Appendix E

**Performance metric on full test set (for all tasks) before and after adaptation the MAML model**

| test compound (test task) | RMSE on full test set before adaptation | Average RMSE on full test set after adaptation | Average RMSE on full test set after adaptation (with selective layers freezing) | Average RMSE on full test set after adaptation (with replay) | Similarity of the molecule to meta-training examples |
|---|---|---|---|---|---|
| n-octane | 0.8398 | 0.8464 | 0.8545 | 0.8389 | 1.0 |
| cyclopentane | 0.8398 | 1.1827 | 1.2086 | 1.0069 | 1.0 |
| cycloheptane | 0.8398 | 1.0568 | 1.0338 | 1.0146 | 1.0 |
| 1-octyne | 0.8398 | 0.8021 | 0.8742 | 0.7966 | 0.94 |
| 1-pentanol | 0.8398 | 1.0385 | 0.9140 | 0.9579 | 0.93 |
| 1-hexyne | 0.8398 | 0.771 | 0.8673 | 0.7797 | 0.73 |
| 2-propanol | 0.8398 | 1.0533 | 0.8379 | 1.1697 | 0.46 |
| tert-butylethyl ether | 0.8398 | 0.9227 | 0.8690 | 0.8713 | 0.44 |
| diethyl ether | 0.8398 | 1.7785 | 1.3217 | 0.7918 | 0.34 |
| methanol | 0.8398 | 0.8462 | 0.8436 | 0.8449 | 0.28 |
| water | 0.8398 | 1.8404 | 0.8173 | 2.0309 | 0.17 |
| acetonitrile | 0.8398 | 0.8412 | 0.8330 | 0.8389 | 0.17 |

## Appendix F

### Ablation study on architectural changes impact while transitioning from MAML to Reptile model

| test compound (test task) | Average RMSE (MAML model) | Average RMSE (MAML with Reptile architecture model) | Average RMSE (TSA-Reptile with linear scaling model) | Similarity of the molecule to meta-training examples |
|---|---|---|---|---|
| n-octane | **0.5257** | 0.7084 | 0.6404 | 1.0 |
| cyclopentane | **0.3694** | 0.4693 | 0.6500 | 1.0 |
| cycloheptane | 0.7316 | **0.7171** | 0.8574 | 1.0 |
| 1-octyne | **0.3267** | 0.6153 | 0.6043 | 0.94 |
| 1-pentanol | 0.6569 | **0.4636** | 0.5801 | 0.93 |
| 1-hexyne | **0.2486** | 0.4370 | 0.3767 | 0.73 |
| 2-propanol | 1.0399 | 0.5398 | **0.4115** | 0.46 |
| tert-butylethyl ether | 0.4848 | **0.2335** | 0.3618 | 0.44 |
| diethyl ether | 0.6345 | **0.5230** | 0.8449 | 0.34 |
| methanol | 1.0455 | 0.8120 | **0.5033** | 0.28 |
| water | 1.4426 | 0.9510 | **0.4812** | 0.17 |
| acetonitrile | 0.2999 | 0.1643 | **0.1496** | 0.17 |
| full test before f.t. | 0.8398 | 0.8284 | **0.6564** | - |

# Appendix G

## Comparison between MAML, Reptile, and fine-tuned GNN

| test compound (test task) | Average RMSE (MAML with Reptile architecture model) | Average RMSE (fine-tuned GNN with Reptile architecture) | Average RMSE (TSA-Reptile with linear scaling model) | Similarity of the molecule to meta-training examples |
|---|---|---|---|---|
| n-octane | 0.7084 | 0.7576 | **0.6404** | 1.0 |
| cyclopentane | **0.4693** | 0.5960 | 0.6500 | 1.0 |
| cycloheptane | **0.7171** | 0.8860 | 0.8574 | 1.0 |
| 1-octyne | 0.6153 | **0.5195** | 0.6043 | 0.94 |
| 1-pentanol | **0.4636** | 0.5235 | 0.5801 | 0.93 |
| 1-hexyne | 0.4370 | **0.3439** | 0.3767 | 0.73 |
| 2-propanol | 0.5398 | 0.6187 | **0.4115** | 0.46 |
| tert-butylethyl ether | **0.2335** | 0.4800 | 0.3618 | 0.44 |
| diethyl ether | **0.5230** | 0.7437 | 0.8449 | 0.34 |
| methanol | 0.8120 | 0.6743 | **0.5033** | 0.28 |
| water | 0.9510 | 0.6721 | **0.4812** | 0.17 |
| acetonitrile | 0.1643 | 0.1740 | **0.1496** | 0.17 |
| full test before f.t. | 0.8284 | 0.9210 | **0.6564** | - |

# References

(1) Baran, K.; Kloskowski, A. Graph Neural Networks and Structural Information on Ionic Liquids: A Cheminformatics Study on Molecular Physicochemical Property Prediction. *The Journal of Physical Chemistry B* **2023**, *127*, 10542–10555.

(2) Heid, E.; Greenman, K. P.; Chung, Y.; Li, S.-C.; Graff, D. E.; Vermeire, F. H.; Wu, H.; Green, W. H.; McGill, C. J. Chemprop: a machine learning package for chemical property prediction. *Journal of Chemical Information and Modeling* **2023**, *64*, 9–17.

(3) Paszke, A. et al. PyTorch: An Imperative Style, High-Performance Deep Learning Library. Advances in Neural Information Processing Systems. 2019.

(4) Fey, M.; Lenssen, J. E. Fast graph representation learning with PyTorch Geometric. *arXiv preprint arXiv:1903.02428* **2019**,
